# Supplementary material for: Proximity proteomics reveals the molecular architecture of phytochrome B photobodies in Arabidopsis thaliana
Source: bioRxiv. 2025 Nov 11:2025.11.09.687487. Preprint. [Version 1] doi: 10.1101/2025.11.09.687487 (PMC12642502; doi:10.1101/2025.11.09.687487)
Supplement: 1 [file NIHPP2025.11.09.687487v1-supplement-1.pdf]

bioRxiv preprint doi: https://doi.org/10.1101/2025.11.09.687487; this version posted November 11, 2025. The copyright holder for this preprint (which was not certified by peer review) is the author/funder, who has granted bioRxiv a license to display the preprint in perpetuity. It is made available under aCC-BY 4.0 International license.

Figure S1. phyB-miniTurbo does not affect phyA-mediated far-red light signaling.

(a) Representative images of 4-day-old Col-0, *phyA-211/phyB-9* (*phyA/phyB*), *PBmTb #3-1, PBmTb #10-1*, and *mTb #2-32* seedlings grown in 10 µmol m^{-2} s^{-1} FR light. (b) Box-and-whisker plots showing hypocotyl lengths of seedlings shown in (a). Boxes indicate the 25th to 75th percentiles with median values shown as horizontal lines; whisker extends to the minimum and maximum values. Different letters represent significant differences (one-way ANOVA with Tukey's HSD test, $P < 0.01$).

Figure S2. Morphological and transcriptional characterization of PBmTb seedlings during de-etiolation.

(a) Representative images of PBmTb #3-1 seedlings transferred from darkness to light for the indicated durations (8, 16, and 24 hour) and under continuous red light (R_C). (b) Transcript levels of plastid-encoded photosynthetic genes *psbA* and *rbcL* relative to *PP2A*. Data represent means ± SD from three biological replicates. Different letters indicate statistically significant differences (one-way ANOVA with Tukey's HSD test, $P < 0.01$).

bioRxiv preprint doi: https://doi.org/10.1101/2025.11.09.687487; this version posted November 11, 2025. The copyright holder for this preprint (which was not certified by peer review) is the author/funder, who has granted bioRxiv a license to display the preprint in perpetuity. It is made available under aCC-BY 4.0 International license.

Figure S3. Quality assessment and phyB enrichment in the PBmTb proximity proteomics dataset.

(a) Principal component analysis (PCA) of proteomic datasets from PBmTb #3-1 and mTb control seedlings showing distinct clustering by genotype across three biological replicates. (b) Abundance of phyB (UNIPROT ID: P14713) detected in PBmTb and mTb samples before and after normalization. Detection of phyB reflects self-biotinylation of the PBmTb fusion protein, confirming active miniTurbo ligase and efficient recovery of the bait protein.
